# Image-Based Plant Disease Identification by Deep Learning Meta-Architectures

**DOI:** 10.3390/plants9111451

**Published:** 2020-10-27

**Authors:** Muhammad Hammad Saleem, Sapna Khanchi, Johan Potgieter, Khalid Mahmood Arif

**Affiliations:** 1 Department of Mechanical and Electrical Engineering, School of Food and Advanced Technology, Massey University, Auckland 0632, New Zealand; H.Saleem@massey.ac.nz (M.H.S.); Sapna.Sapna.1@uni.massey.ac.nz (S.K.); 2 Massey Agritech Partnership Research Centre, School of Food and Advanced Technology, Massey University, Palmerston North 4442, New Zealand; J.Potgieter@massey.ac.nz

**Keywords:** deep learning, plant disease detection, transfer learning, optimization algorithms, mean average precision

## Abstract

The identification of plant disease is an imperative part of crop monitoring systems. Computer vision and deep learning (DL) techniques have been proven to be state-of-the-art to address various agricultural problems. This research performed the complex tasks of localization and classification of the disease in plant leaves. In this regard, three DL meta-architectures including the Single Shot MultiBox Detector (SSD), Faster Region-based Convolutional Neural Network (RCNN), and Region-based Fully Convolutional Networks (RFCN) were applied by using the TensorFlow object detection framework. All the DL models were trained/tested on a controlled environment dataset to recognize the disease in plant species. Moreover, an improvement in the mean average precision of the best-obtained deep learning architecture was attempted through different state-of-the-art deep learning optimizers. The SSD model trained with an Adam optimizer exhibited the highest mean average precision (mAP) of 73.07%. The successful identification of 26 different types of defected and 12 types of healthy leaves in a single framework proved the novelty of the work. In the future, the proposed detection methodology can also be adopted for other agricultural applications. Moreover, the generated weights can be reused for future real-time detection of plant disease in a controlled/uncontrolled environment.

## 1. Introduction

In agricultural crops, leaves play a vital role to provide information about the amount and nature of horticultural yield. Several factors affect food production such as climate change, presence of weed, and soil infertility. Apart from that, plant or leaf disease is a global threat to the growth of several agricultural products and a source of economic losses [1]. The failure to diagnose infections/bacteria/virus in plants leads subsequently to insufficient pesticide/fungicide use. Therefore, plant diseases have been largely considered in the scientific community, with a focus on the biological features of diseases. Precision farming uses the most advanced technology for the optimization of decision-making. The visual inspections by experts and biological review are usually carried out through plant diagnosis when required. This method, however, is typically time-consuming and cost ineffective. To address these issues, it is necessary to detect plant diseases by advanced and intelligent techniques.

To perform the agricultural operations, conventional machine learning (ML) algorithms have been applied in many studies [2,3]. However, recently, deep learning (DL) as a sub-set of ML, has been strikingly effective for real-life object detection, recognition, and classification purposes [4,5,6]. Therefore, agricultural research has been moving towards the DL-based solutions. The DL techniques have been accomplished state-of-the-art results to perform the agricultural operations including crop/weed discrimination [7,8], fruit harvesting [9,10], and plant recognition [11,12,13,14]. Similarly, recent studies have also focused on another important agricultural issue of plant disease identification [6].

Several state-of-the-art DL models have been applied to perform plant disease classification by using well-known DL architectures. Moreover, some researchers introduced modified versions of DL algorithms to improve the performance of the classification of disease in several plant species. A few of the prominent/recent studies are highlighted in this section. For example, a recent article presented a comparative analysis of various Convolutional Neural Networks (CNN) and DL optimizers to attain better results of plant disease classification [15]. A study proposed a CNN model to classify disease in tea leaves [16]. Another study was conducted to propose two revised versions of MobileNet models for the classification of several plant diseases [17]. A recent article presented two deep learning architectures based on residual learning and attention methods to classify tomato leaf diseases and achieved a higher overall accuracy [18]. Another CNN-based architecture was proposed to classify disease in the PlantVillage dataset, and it performed better than the well-known DL models including AlexNet, VGG-16, Inception-v3, and ResNet [19]. A recent article proposed a CNN-based model for the classification of groundnut disease [20]. Similarly, few studies focused on the advanced training techniques; for example, [21] evaluated the performance of AlexNet and GoogLeNet trained from scratch and transfer learning approaches. A comparative study was conducted to show the significance of the fine-tuning technique by comparing state-of-the-art DL architectures for the classification of plant disease [22]. More recent developments regarding the specific task of plant disease classification are comprehensively presented in [6,15].

To address the task of object identification, the classification and localization of objects are performed in a single platform by using deep learning meta-architectures. In this regard, few DL algorithms have been developed. The Region-based Convolution Neural Network (RCNN) was among the first modern techniques towards image detection tasks through CNN [23]. Afterward, the successful implementation of regional proposal methods proved significant developments in object identification. In the context of plant disease recognition, very few studies have been conducted to perform this complex agricultural operation by DL techniques. For example, in [24], the deep learning models were implemented to perform plant disease localization and diagnosis. The authors used their own annotated images of tomato leaf and successfully obtained a higher mean average precision. In [25], two different approaches were developed and compared to perform automated pest detection based on ML/DL learning strategies. This work focused on the detection of the harmful pest in greenhouse tomato and pepper crops. Their findings showed that the deep learning methods provided a better result as compared to the machine learning algorithms due to its capability to perform detection and classification tasks in one step. A recent article presented the DL approach to diagnose disease in Cassava leaves by using the Single Shot MultiBox Detector (SSD) and achieved satisfactory results [26]. Another recent research considered the plant disease recognition task by CNN to estimate the severity of defects in the plant leaves [27].

From the literature, it can be concluded that most of the recent researches have been focused on the task of plant disease classification (only classify the type of disease among several plant species). However, the complex task of plant disease identification (both localization and classification of the disease in the plant) has been given very little attention. Moreover, none of the previous approaches has performed a comprehensive study regarding the detection/identification of 38 classes of plant disease by advanced DL meta-architectures. Therefore, in this research, an evaluation of three successful DL-based object detection techniques including the Single Feed-forward Neural Network, Region Proposal Network, and Region-based Fully Convolutional Network has been carried out using a transfer learning technique that focused on an important agricultural problem of plant disease identification. The transfer learning technique is applied due to its successful performance for many object recognition tasks. From the practical point of view, reuse or transfer of information from previously learned tasks for learning new tasks increases the accuracy of the DL architectures. In this research, we have shown the final ConvNet checkpoints of the detection tools. Moreover, recently, the research community is focusing on better optimization of weight parameters of neural networks [15]. Thereby, in this work, the performance of three state-of-the-art deep learning optimizers was also analysed, which significantly improved the prediction ability (true positive detection rate) of top selected DL meta-architectures.

The main contributions of this research are summarized as follows:

1. A comprehensive study of deep learning meta-architectures has been conducted for the identification of disease in several plant species infected by fungi, infection, virus, and bacteria.
2. An attempt has been made towards the improvement in the performance of DL meta-architectures specifically for plant disease recognition/identification tasks by using three different state-of-the-art DL optimization methods including Stochastic Gradient Descent (SGD) with Momentum, Adaptative Moment Estimation (Adam), and Root Mean Square Propagation (RMSProp).
3. The weights obtained after the training of the DL models could also be used for the other datasets related to plant disease.

The rest of the article is presented as follows: Section 2 explains the overall methodology, applied framework, selection of datasets, annotation of dataset images, DL meta-architecture, DL optimizers, experimental setup, and performance metric. Section 3 presents the performance of all the DL methods along with the improvement in their performance by optimization algorithms, and Section 4 provides the conclusion with some future works.

## 2. Materials and Methods

**Figure 1:**
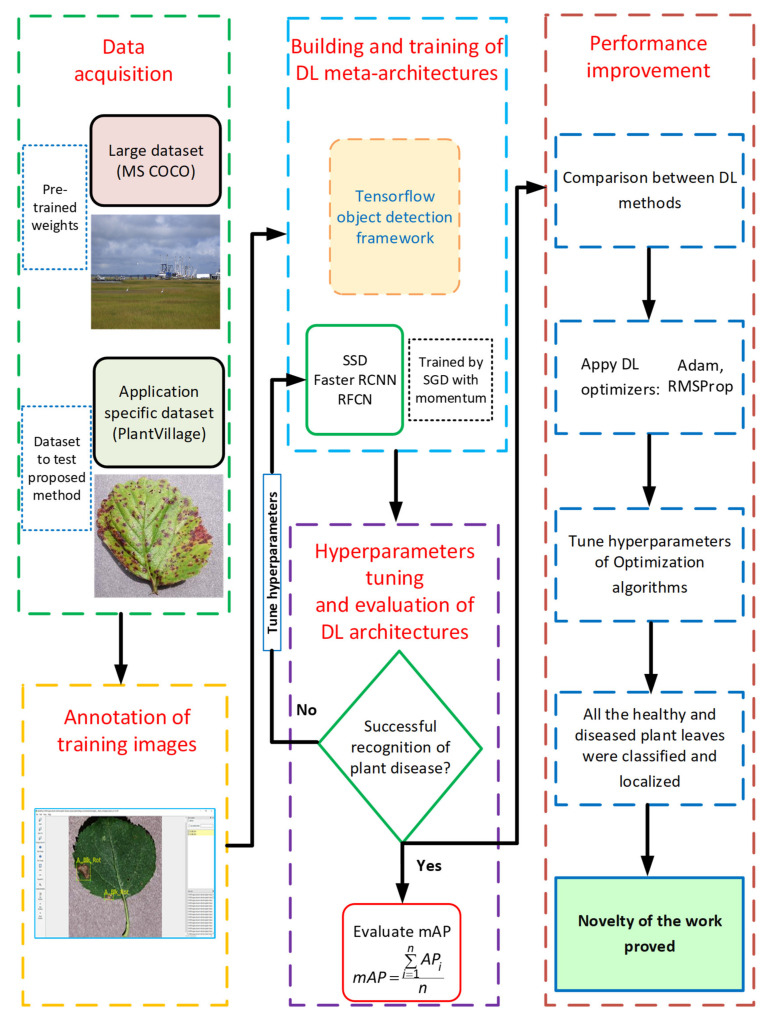
The flow diagram of this research.

This article addresses the plant disease identification task by state-of-the-art three deep learning meta-architectures prominently Faster Region-based Convolutional Neural Network (RCNN), Single Shot MultiBox Detector (SSD), and Region-based Fully Convolutional Networks (RFCN). The overall methodology for this research is presented in Figure 1. The first step was the selection of two datasets: a large dataset to obtain the pre-trained weights for transfer learning, and the second dataset was related to the different classes of disease on plant leaves. The next step was the annotation of the training dataset by an online available tool called LabelImg. This led to constructing and training the DL architectures. Then, the recognition of all the classes of plant disease was checked to tune the hyperparameters of the SGD optimizer. Next, the performance of the learned neural networks was evaluated on the images of the testing (unseen) dataset. Here, the actual outputs were compared with the expected outputs to identify errors. Furthermore, the mean average precision (mAP) of all the neural networks was measured to obtain the best suited DL model. The further improvement in the mAP was proposed by using various DL optimizers. Finally, the proposed method was successful to classify and localize the healthy/diseased leaves of various plant species.

### 2.1. Generalized Framework

**Figure 2:**
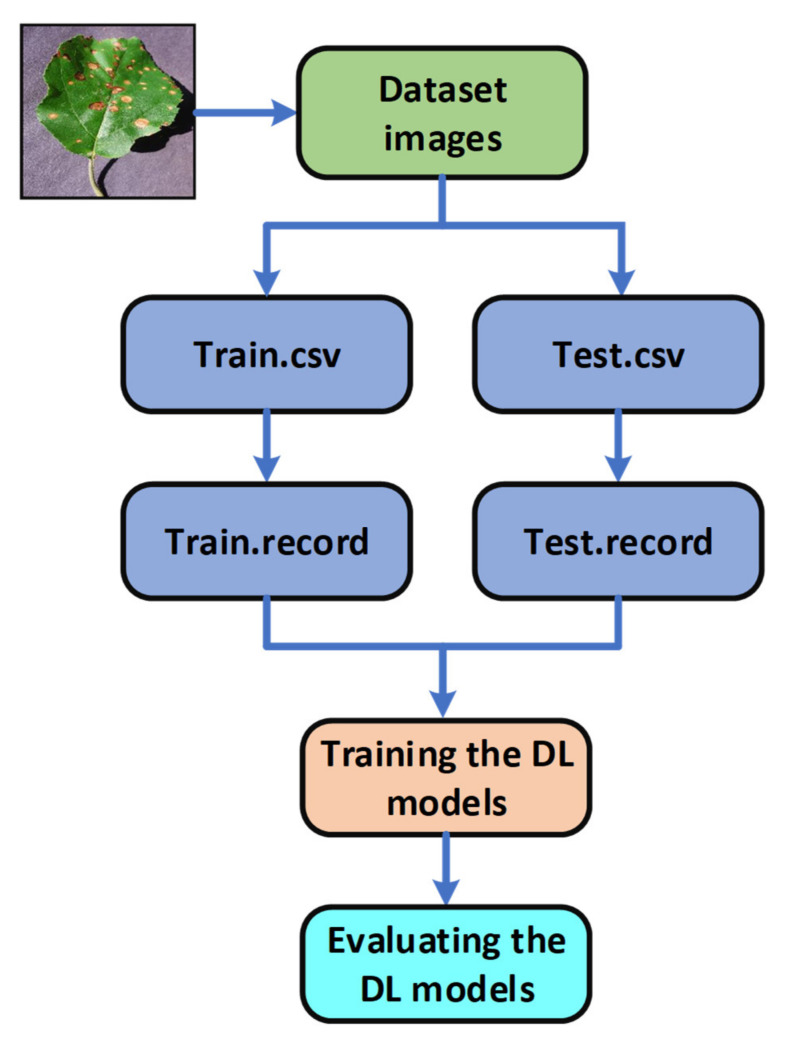
Generalize framework to train and test the deep learning (DL) meta-architectures for plant disease identification.

The generalized framework of training and testing the DL models is presented in Figure 2 which consists of dataset images having their corresponding XML files. The XML data were converted into CSV format. Then, TF records from the CSV files were generated, as TensorFlow accepts the TF format of the data to feed into the network while training the DL architectures. The DL detectors were constructed by taking training images with bounding box coordinates and then evaluated their performance on the testing dataset.

### 2.2. Dataset Selection

Few datasets have been developed and used for various real-life operations consisting of a huge number of classes. For example, in object classification/detection research, the dataset of ImageNet [28], which includes unprecedented numbers of images, has recently made breakthroughs. Similarly, the MS COCO dataset [29] consists of 91 common object classes with 82 of these having more than 5k labelled instances. A total of 2500k data instances are labelled in 328k pictures. The MS COCO dataset contains substantially more object instances per picture (7.7) as compared to the ImageNet (3.0) and PASCAL (2.3) datasets. Therefore, we used the training weights of the MS COCO dataset for the transfer learning purpose. Next, the PlantVillage dataset [30] was selected, as it contains images that are relevant to the area of interest. This dataset consists of images of 14 plant varieties. The dataset shows 17 fungal infections, 4 bacterial diseases, 2 fungal illnesses, 2 infectious diseases, and 1 mite-induced disease [30]. Twelve plant species also show images of healthy leaves that have no obvious illness.

### 2.3. Annotation of the Training Dataset

**Figure 3:**
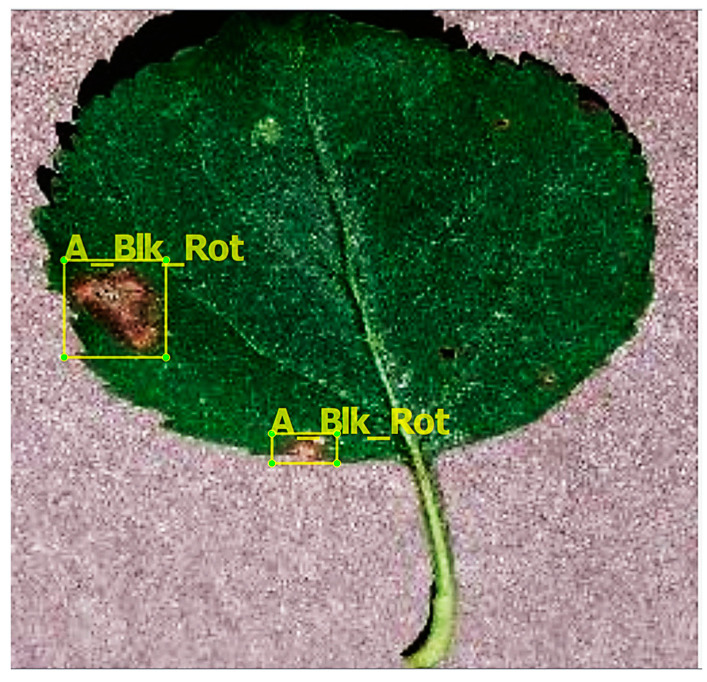
An example of an annotated image using the LabelImg tool.

The PlantVillage dataset was divided into three sub-datasets: 70% (38017 images) for training, 20% (10858 images) for validation, and 10% (5431 images) for testing [15,17]. Then, the annotation of training dataset images was the first step towards the plant disease identification task by DL meta-architectures. In this study, the training images were annotated by LabelImg, which is an open-source graphic image annotation application. As a result, the bounding box coordinates (*Xmin*, *Ymin*, *Xmax*, and *Ymax*) were created. These bounding boxes are the ground truth boxes that evaluate as the intersection of the union (IoU) with the prediction bounding box. To save annotations as XML files, the Pascal VOC format was used. An example of an annotated dataset image is given in Figure 3. Table 1 shows the details regarding the classes of the PlantVillage dataset.

### 2.4. Deep Learning Meta-Architectures

In this research, three successful DL meta-architectures were considered for the detection of plant disease. These models consist of a base network and a feature extractor. The following sub-sections provide an insight of these DL architectures to elaborate their functionality for performing an image recognition/identification task along with the overall loss function of the respective models.

#### 2.4.1. Single Shot MultiBox Detector (SSD)

**Figure 4:**
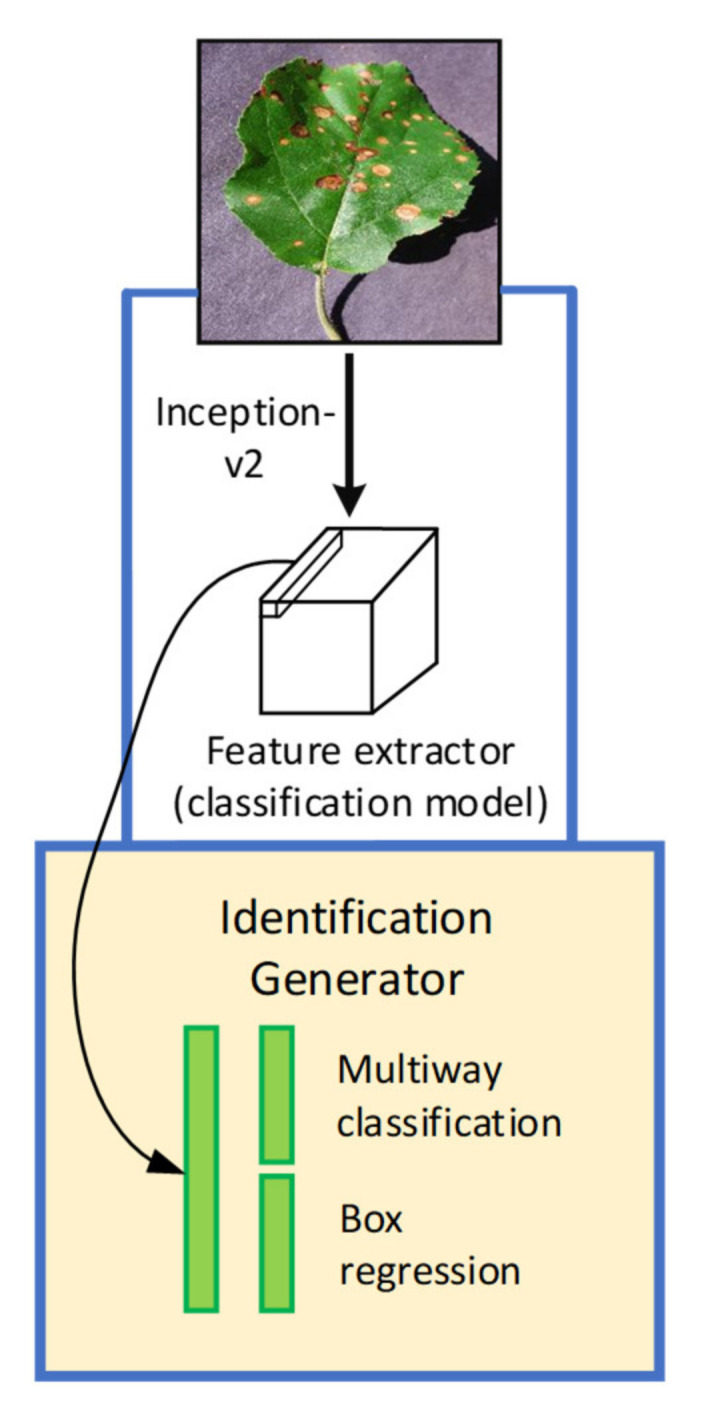
The basic architecture of the Single Shot MultiBox Detector (SSD) [32].

The SSD model is simple due to the elimination of the region proposal and subsequent pixel or resampling of features. This DL model includes all computations in one network, which is why it is known as a single-shot detector [31]. Experimental findings on the MS COCO, ILSVRC, and PASCAL VOC datasets revealed that the SSD achieved comparatively better precision than the other DL models such as Faster RCNN, and much faster computation time while providing a unified training and inference framework [31]. The key feature of SSD is the use of small convolution filters, such as 4x4 and 8x8; feature maps for category score; and box offset prediction for the collection of default bounding boxes. The conceptual diagram of the SSD model is presented in Figure 4. The overall loss for SSD architecture is evaluated by Equation (1) [31]:

(1) $$L(x,c,l,g)=\frac{1}{N}[L_{conf}(x,c)+\alpha L_{loc}(x,l,g)]$$

where, *N*, *L_conf_*, *α*, and *L_los_* represent the number of matched default boxes, confidence loss, weight term, and localization loss, respectively.

#### 2.4.2. Faster Region-based Convolutional Neural Network (Faster-RCNN)

**Figure 5:**
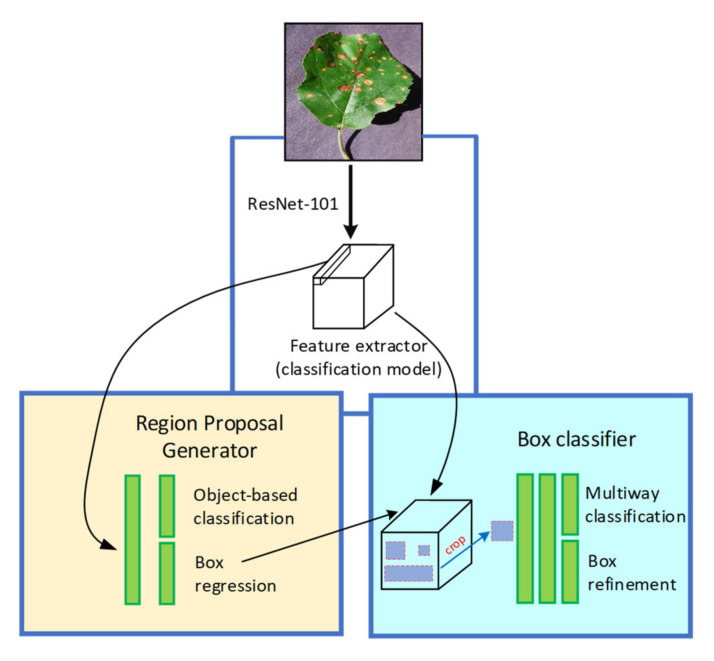
The basic architecture of the Faster Region-based Convolutional Neural Network (RCNN) [32].

In Faster RCNN architecture, the object detection task performs at two different stages as compared to SSD. At the region proposal network (RPN) stage, the images are processed to generate region proposals directly through feature extractors (Inception and ResNet) instead of an external algorithm such as Edge Boxes. These features are used to forecast class-specific proposals for each intermediate convolutional layer. Then, the generated anchor boxes are used at the second step of detecting the characteristics of the same immediate layer of an image. Figure 5 presents the basic concept of the Faster RCNN model, and the overall loss is evaluated by Equation (2) [33]:

(2) $$L[\{p_{i}\},\{t_{i}\}]=\frac{1}{N_{cls}}\sum_{i}L_{cls}(p_{i},p_{i}{}^{*})+\lambda \frac{1}{N_{reg}}\sum_{i}p_{i}{}^{*}L_{reg}(t_{i},t_{i}{}^{*})$$

where *i* indicates the index of anchor (responsible for obtaining bounding boxes of various sizes/ratios and used as a reference while predicting object locations); *p_i_*, p_i_^*^, *t_i_*, *N_cls_*, *λ*, *L_cls_*, *L_reg_*, and *N_reg_* present the output score from classification branch for anchor *i*, ground truth label (0/1), output prediction of the regressor layer (which consists of 4 variables (*t_x_*, *t_y_*, *t_w_*, *t_h_*)), number of anchors in mini-batch, balancing parameter, classification loss, the regressor loss (it actuates only if anchor contains an object that is ground truth (p_i_^*^) is 1), and number of anchors in mini-batch, respectively. Here, t_i_^*^ is the ground truth box with a positive anchor.

#### 2.4.3. Region-based Fully Convolutional Networks (RFCN)

**Figure 6:**
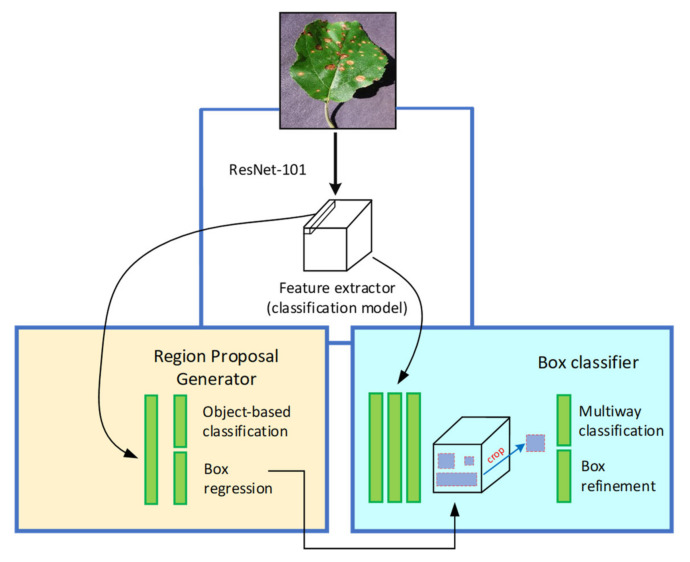
The basic architecture of the Region-based Fully Convolutional Network (RFCN) [32].

This network is much like the Faster-RCNN, except for the removal of fully convolutional layers after the region of interest (ROI) pooling. After ROI pooling, the region proposals generate the same set of score maps for average voting. Moreover, this DL architecture has a lesser complexity level because there is no learnable layer after ROI which significantly reduces its computation time as compared to the models such as Faster RCNN. Figure 6 presents the basic concept of RFCN architecture with its corresponding proposal generator and feature extractor. The overall loss expression can be seen in Equations (3) and (4) [34]:

(3) $$L(s,t_{x,y,w,h})=L_{cls}(s_{c}{}^{*})+\lambda [c^{*}>0]L_{reg}(t,t^{*})$$

where L_cls_ (s_c_^*^) is a cross-entropy loss for classification and calculates by:

(4) $$L_{cls}=-log\left(s_{c}{}^{*}\right)$$

In Equation (3), *λ* is the balance weight, which is set to 1; [c^*^ > 0] is an indicator, which is equal to 1 if the argument is true and 0 otherwise. L_reg_(t,t^*^) is the bounding box regression loss and evaluated by *smooth_L1_* function, c^*^ indicates the ground-truth label of the region of interest (ROI), and t^*^ presents ROI’s ground truth box.

### 2.5. Deep Learning Optimizers

This article also attempted an improvement in the performance of DL meta-architectures by using various deep learning optimizers. The basic concept of these optimizing functions along with the mathematical details are presented in the following sub-sections.

#### 2.5.1. Stochastic Gradient Descent (SGD) with Momentum

The gradient descent is the most widely used optimization algorithm for neural networks [35]. Its momentum version has faster convergence ability than the standard algorithm. The basic idea is to calculate the exponentially weighted average of the gradients and use the gradients to update the weights. To optimize the cost function, gradient descent slowly oscillates the loss towards the minimum, this slows down gradient descent and avoids by a large learning rate. However, if a larger learning rate is used, then it might end up with problems such as overshooting and diverging output. In contrast with the SGD optimizer, which used *dw* (calculated gradient of the weights) and *db* (calculated gradient of the biases) independently, the exponentially weighted averages of *dw* and *db* are taken for the momentum algorithm by the following equations (Equations (5) and (6)):

(5) Vdw=β*Vdw+(1−β)*dw

(6) Vdb=β*Vdb+(1−β)*db

where, *β* indicates the momentum that should be higher to smooth the update, and its default value is 0.9. *Vdw* and *Vdb* are weighted averages of optimization parameters weights and biases, respectively. After obtaining the exponentially weighted averages, weights and biases are updated by (Equations (7) and (8)):

(7) W=W−lr*Vdw

(8) b=b−lr*Vdb

where *lr*, *W* and *b* are learning rate, weight, and bias, respectively.

#### 2.5.2. Root Mean Square Propagation (RMSProp)

The RMSProp optimization algorithm [36] limits the oscillations that generate during the loss optimization in the direction of bias, which helps to consider a larger learning rate without having an overshooting problem in training of the model. The difference between momentum and RMSProp lies in the calculation of their gradients, and weight/bias updates as shown below (Equations (9)–(12)):

(9) $$Vdw=\beta *Vdw+(1-\beta )*dw^{2}$$

(10) $$Vdb=\beta *Vdb+(1-\beta )*db^{2}$$

(11) $$W=W-lr*\frac{dw}{\sqrt{vdw}+\xi }$$

(12) $$b=b-lr*\frac{db}{\sqrt{vdb}+\xi }$$

The *Vdw* of the RMSProp optimizer could be relatively small (even 0); therefore, epsilon (𝜉) adds in the denominator for numerical stability. When *Vdw* is relatively small, it increases weights *(W)*, and then the updates in the direction of weights become fast. However, *Vdb* is relatively large, which decreases bias *(b)* to slow down the updates in its direction.

#### 2.5.3. Adaptive Moment Estimation (Adam)

The idea behind the Adam optimization algorithm is taking momentum and RMSProp and putting them together [37]. It means that the Adam optimizer retains an exponentially decaying average gradient of the previous gradients as well as previously squared gradients. First, the initialization of *Vdw*, *Vdb*, *Sdw* (element-wise squaring of *Vdw*), and *Sdb* (element-wise squaring of *Vdb*) is set to zero. Then, for a certain number of iterations, this algorithm computes the *dw* and *db* using current mini-batch and performs exponentially weighted average by using Equations (5), (6), (9), and (10):

Then, the calculations for the corrected *Vdw*, *Vdb*, *Sdw,* and *Sdb* are performed for bias correction by the following equations (Equations (13)–(16)):

(13) $$Vdw^{corrected}=Vdw/(1-\beta_{1}t)$$

(14) $$Vdb^{corrected}=Vdb/(1-\beta_{1}t)$$

(15) $$Sdw^{corrected}=Sdw/(1-\beta_{2}t)$$

(16) $$Sdb^{corrected}=Sdb/(1-\beta_{2}t)$$

where, *β_1_* and *β_2_* indicate exponential decay rate for the first moment and second moment, respectively.

Weight (W) and bias (b) are updated by (Equations (17) and (18)):

(17) $$W=W-lr\frac{Vdw^{corrected}}{\sqrt{Sdw^{corrected}}+\xi }$$

(18) $$b=b-lr\frac{Vdb^{corrected}}{\sqrt{Sdb^{corrected}}+\xi }$$

### 2.6. Experimental Setup

The experiments are based on three popular DL meta-architectures: SSD, Faster-RCNN, and R-FCN, which were previously trained on 1.5 million images (80 categories) of the Common Objects in Context (COCO) dataset. The transfer learning technique was used to obtain better detection results. First, the trained layers were frozen to reuse some fundamental features such as corners, borders, and edges; then, a few new and workable layers were added that learned the specific features of the new dataset (PlantVillage). The backbone architectures named Inception-v2 [38], Inception ResNet-v2 [39], and versions of ResNet including ResNet-50 and ResNet-101 [40] were used with the base networks to classify and localize the plant disease. Table 2 presents the base networks with feature extraction methods along with their performance measured in mAP on the COCO dataset.

Table 3 presents the hyperparameters: *α* (learning rate), *β_1_* known as the first moment has a default value of 0.9_,_ *β_2_* known as the second moment has a default value of 0.999, and epsilon (𝜉)= 10^−8^. However, the default value of 𝜉 may not be sufficient in general for some machine learning problems. The best learning rate was selected from three sets (10^−4^, 10^−5^, 10^−6^) to determine the efficacy of the initializations. These learning rates were considered for different steps of iterations while training [31,33,37]. The tuning of the hyperparameters of all the DL optimizers was performed using the random search technique [15,41]. All the DL models were trained using Graphics Processing Units (NVIDIA GTX 1650 and 1050) for high-performance acceleration.

### 2.7. Performance Metric

**Figure 7:**
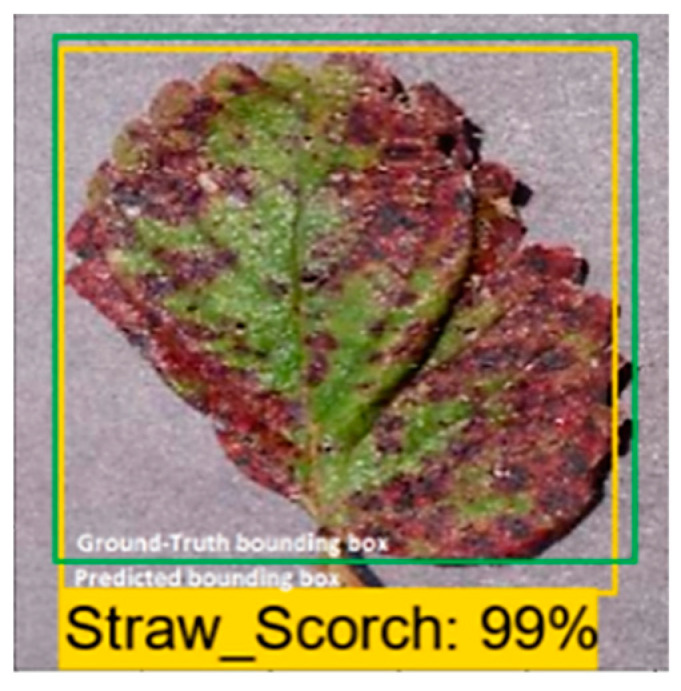
Visual example of ground truth bounding box (green) versus predicted bounding box (yellow) for intersection of the union (IoU) metric.

The performance of the DL-based plant disease detectors was evaluated by using mean average precision (mAP). This performance metric is commonly used with the DL meta-architectures (SSD, Faster-RCNN) to detect artifacts such as COCO [29] and PASCAL’s VOC challenge [42]. Any algorithm providing the predicted bounding boxes as an output can be assessed with intersection of union (IoU), Average Precision (AP), and mAP [42]. The x, y coordinates require (*Xmin, Xmax, Ymin,* and *Ymax*) to track the efficiency of the DL architectures. In Figure 7, an image belongs to a strawberry leaf class is presented, where the DL model provided an output in the form of a predicted bounding box with scorch disease on the image. Two bounding boxes can be seen in Figure 7: one shows the exact location of the healthy/defected part in an image, named as the ground-truth bounding box. Another is an actual predicted bounding box that is drawn by the trained DL model.

For evaluating the mAP, it should be noted that the precision measures how accurate the predictions are—that is, the percentage of the correct predictions—and recall measures how well all positive outcomes are found. The average precision (AP) was assessed with an 11-point interpolated average precision method. The precision and recall were computed for each class. The AP is the average precision across all unique recall levels. Before measuring AP, we first interplay the precision at multiple recall levels. At a certain recall level *r*, the interpolated precision (*p_int_*) is specified as the highest precision for a recall level *r′ ≥ r* [24,42] (Equations (19) and (20)).

(19) $$p_{\mathrm{int}}(r)=\max p(r^{\prime })$$

where *p(r′)* is the measured precision at the max recall *r*$r^{\prime }$.

The AP is then described as the mean precision at the eleven recall rates equally spaced [0, 0.1, 0.2, ......, 1].

(20) $$AP\;=\;\frac{1}{11}\sum_{r\in \left\{0,0.1\;,\;0.2,\;.....1\right\}}p_{\mathrm{int}}(r)$$

The mean average accuracy (mAP) is the approximate average value of all individual APs. To evaluate the mAP, the AP of each class was first calculated (as described above). Then, the mAP was found by Equation (21):

(21) $$mAP=\;\frac{\sum_{i=1}^{n}AP_{i}}{n}$$

where n = 38 (number of classes).

## 3. Results and Discussion

The goal of this research is not only the identification of the presence of diseased and healthy leaves but also to locate a confidence score indicating the likelihood that there is a correct (true positive) class in a bounding box. The score was considered between 0 and 1 (or 0–100%), indicating how much precisely the type of plant disease was recognized. It was empirically observed that all the DL meta-architectures required 126 epochs (200,000 iterations) to converge their training. The loss plots of each DL architecture with its detection results are presented in this section. Moreover, the improvement in the performance of the best-suited architectures is also presented. The mAP attained by each DL meta-architecture with its corresponding optimizer is shown in Table 4.

### 3.1. Performance of Deep Learning Meta-Architectures

During the first phase of the proposed approach, all the DL architectures were trained with the momentum optimizer due to its fast convergence ability [43]. The SSD model outperformed Faster RCNN and RFCN models. Further explanations of the results of each model are provided as follows:

#### 3.1.1. SSD Architecture

**Figure 8:**
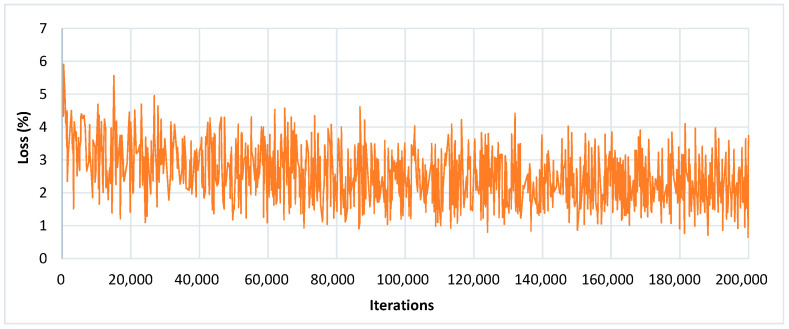
Training loss curve of SSD with Inception-v2 model.

**Figure 9:**
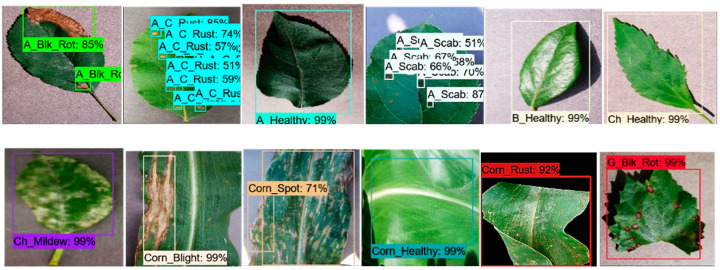

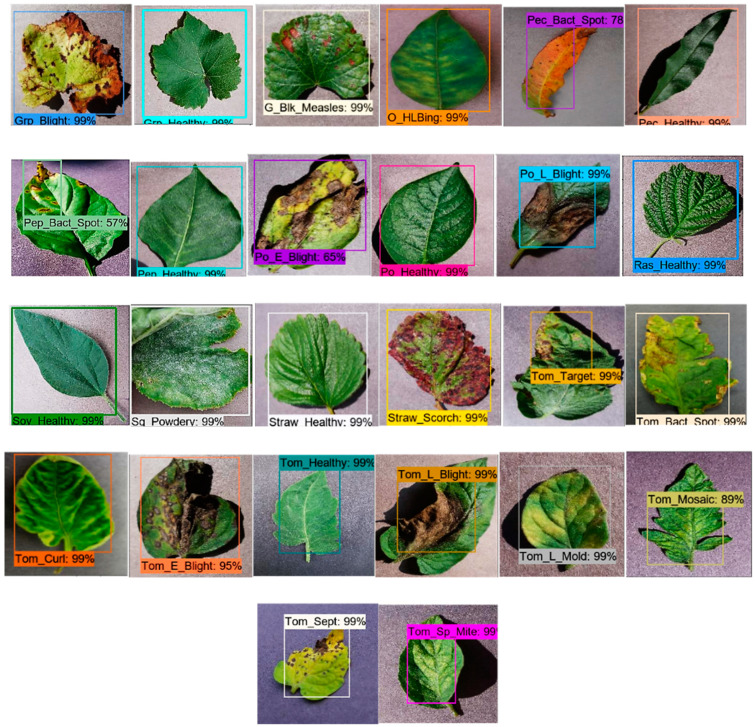
The detection results by the SSD with the Inception-v2 model having a threshold score of 0.5. Each predicted box is associated with a category label having a confidence score between 0 and 100%.

An input image of 300 × 300 size was considered for all the experiments. The SSD architecture was trained with the feature extraction method called Inception-v2 with different learning rates. The model was trained by using SGD with the momentum optimizer using the learning rate as 3 × 10^−4^, 3 × 10^−5,^ and 3 × 10^− 6^ for 90k, 30k, and 80k iterations, respectively. This model took approximately 4.25 days to complete its training. The training loss curve of the SSD model is shown in Figure 8. At the end of the training, the loss curve indicated its fluctuation between 0.64% and 3.73%. After the training of the SSD model, the images from the testing dataset were used to classify and localize the defected spots of plant disease. Figure 9 shows the detection results obtained by the SSD along with their confidence score. Table 4 indicates the average precision of each leaf category and the mAP of 38 leaf disease categories. The mAP obtained by this state-of-the-art DL architecture was 66.51%, which is the highest among all the other models. It is noticed that the results of six plant classes such as blueberry healthy, grape healthy, grape black measles, strawberry healthy, tomato healthy, and tomato curl virus were quite promising, due to their 100% average precision. For around 12 leaf categories, the average precision was more than 90%. Around 14 disease classes achieved low precision (less than 50%). It is also noticed that the precision of corn gray leaf spot was the lowest among all the other classes of plant disease, which was addressed in the next step of the proposed method.

#### 3.1.2. Faster-RCNN Architecture

**Figure 10:**
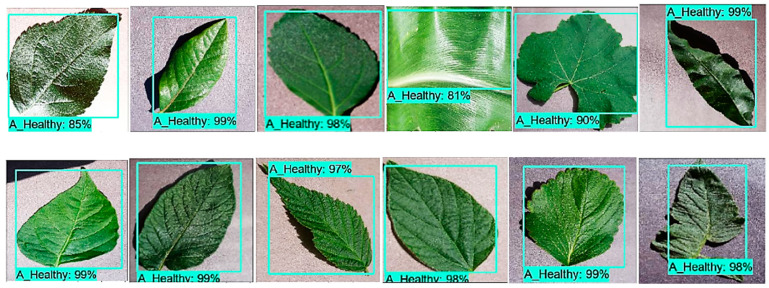
False positive detection results of healthy classes by Faster-RCNN with ResNet-50 architecture. The first image is from the apple healthy category, which is the only class that the model detects correctly. Other images were originally from blueberry, cherry, corn, grape, peach, pepper bell, potato, raspberry, soybean, strawberry, and tomato healthy classes, but the model identified them as Apple healthy class.

**Figure 11:**
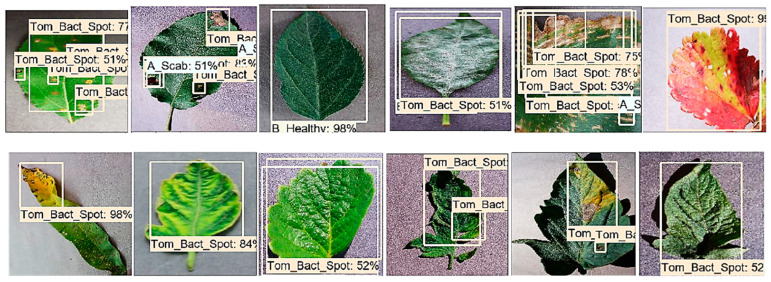
Some of the examples of false positives detection results using the Faster-RCNN with Inception ResNet-v2 architecture. It identified most of the leaf categories as the tomato bacterial spot.

The Faster-RCNN model was trained with the feature extractors including ResNet-50, ResNet-101, Inception-v2, and Inception ResNet-v2. All the feature extraction methods were trained with momentum optimizer using learning rate 3 × 10^−4^, 3 × 10^−5,^ and 3 × 10^−6^ for 90k, 30k, and 80k iterations, respectively. Initially, the Faster RCNN model was trained with the feature extraction methods such as ResNet-50 and Inception Resnet-v2, but they failed to detect and localize most of the classes (as shown in Figure 10, Figure 11, and Table 4) and resulted in the lowest mAP among all the DL architectures. This was due to the presence of some challenging disease categories (potato early blight, potato late blight, tomato early blight, and tomato late blight, etc.), since specific features such as leaf shape, disease spots, and colour of disease spot were quite similar.

**Figure 12:**
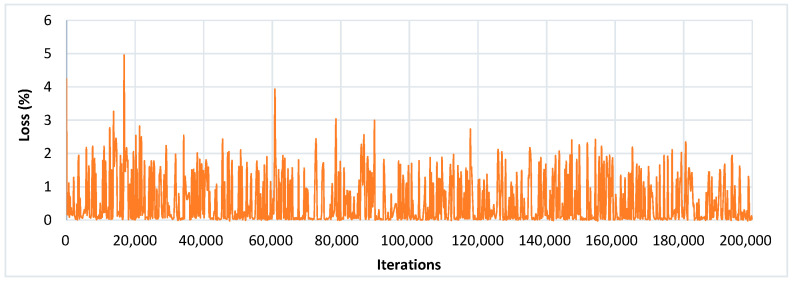
Training loss curve of Faster-RCNN with ResNet-101 model.

To obtain a noticeable improvement in the identification results by the Faster-RCNN model, two other classification models/feature extractors (Inception-v2 and ResNet-101) were also considered. Due to different feature extractors, the training time of the Faster RCNN model was varied. For example, ResNet-50 required the lowest training time of around 34 h, whereas Inception-v2 was the slowest among the other classification models as it took approximately 48 h to complete its training. However, ResNet-101 and Inception ResNet-v2 needed 37.5 and 44.20 h, respectively. It was observed that the baseline Faster-RCNN performed well when combined with the feature extraction method ResNet-101. It achieved 60.92% mAP@0.5, which is 9.73% higher than with the Inception-v2 (51.19%) with the same training settings. The training loss obtained by the Faster RCNN with the ResNet-101 model is presented in Figure 12, and its lower percentage error proved the effective learning of the specific features of plant disease after 200k iterations. From Table 4, it can be observed that the Faster RCNN with ResNet-101 architecture identified Grape Black Measles disease with 100% precision. Moreover, it attained more than 90% precision for almost 14 leaf classes. However, 13 classes achieved a precision of less than 50%. A further five classes were poorly detected and resulted in less than 10% precision. It is also noticed that two classes (potato healthy and potato late blight) failed to detect by Faster-RCNN with the ResNet-101 model.

#### 3.1.3. R-FCN Architecture

**Figure 13:**
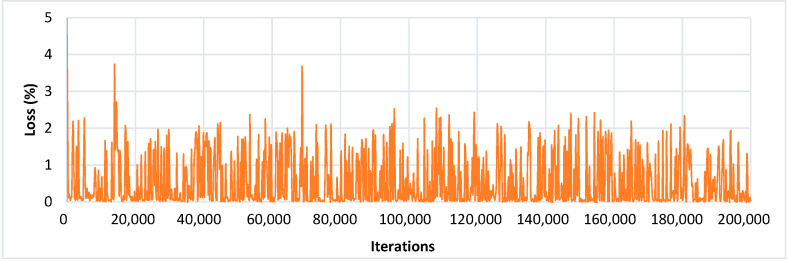
Training loss curve of the R-FCN model.

Using the ResNet-101 as the feature extractor, the RFCN model achieved good detection results of 83.6% mAP on the PASCAL VOC dataset. Therefore, the same feature extractor with the baseline model (RFCN) was also considered in this research. In this DL method, all learnable weight layers are convolutional, which computes the feature map on the entire image. The R-FCN model was fine-tuned and trained with the momentum optimizer using the learning rate 3 × 10^-4^ for 90k steps, and then continued training for the next 30k steps with 3 × 10^-5^, and finally, 80k steps with 3 × 10^− 6^. This model completed 200k iterations in 33.7 h. The training loss obtained by the RFCN model is presented in Figure 13, and its percentage loss oscillated from 0.03% to 1.28% after 200k iterations.

**Figure 14:**
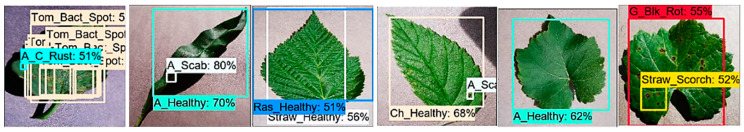
Some examples of false-positive detection results by the RFCN model.

This model identified a lesser number of classes than the SSD and Faster RCNN (with ResNet-101) models; the failed classes were: corn healthy, grape healthy, peach healthy, potato healthy, tomato Septoria, tomato early blight, and tomato late blight (as shown in Table 4). The strawberry scorch class achieved the perfect average precision of 100%. It is also observed that 9 plant classes provided good detection results and achieved considerable precision (more than 90%). Around 11 other classes showed an average precision of less than 10%. Few examples of the false/confused detection results by the RFCN model are shown in Figure 14.

### 3.2. Overall Remarks for SSD, Faster RCNN, and RFCN Architectures

From Table 4, important observations regarding the performance of the DL meta-architectures are presented as follows:

- The SSD model achieved the highest mAP among all the DL meta-architectures. This is due to the structural behaviour of the SSD model which provides a fixed-size predictive box set and scores at each feature-layer position of a kernel. The convolutional layers are added to the last of the base network which predicts multiple scales [31]. The projected performance value boxes in each feature map location compared to the default position boxes are determined using an intermediate connected layer in these positions instead of a fully convolution layer.
- Another significant distinction of the SSD model is that the information in ground-level truth boxes allocates to different outputs within the defined collection of detector outputs during SSD training compared to other regional networks. The structure of the network decides which ground box should be matched with its corresponding default box during the training stage, known as matching strategy in SSD. Thereby, the use of several convolutional bounding box outputs connected to features maps at the top of the network made this model successful as compared to other region-based methods.
- The base network SSD combined with the” Inception” model performed better than the Faster-RCNN combined with the same feature extraction method. Moreover, Table 4 shows that the base network Faster-RCNN with feature extractor ResNet-101 showed relatively higher mAP than with the Inception model.
- The RFCN model achieved lower mAP than the SSD and Faster RCNN (with ResNet-101) architectures.
- More interestingly, the SSD architecture was able to detect few of those classes that were completely undetected by the Faster RCNN and RFCN models (as shown in Table 4).
- Following the proposed methodology presented in Section 2, the SSD with Inception-v2 and Faster RCNN with ResNet-101 models achieved the highest mAP among all the other DL meta-architectures. Therefore, they were selected for the next stage of this research.

### 3.3. Performance Improvement by DL Optimization Algorithms

**Figure 15:**
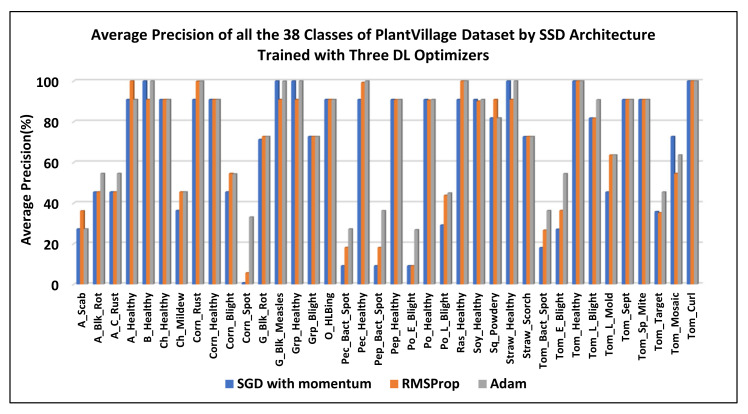
Performance plot of the SSD model trained with three DL optimizers.

**Figure 16:**
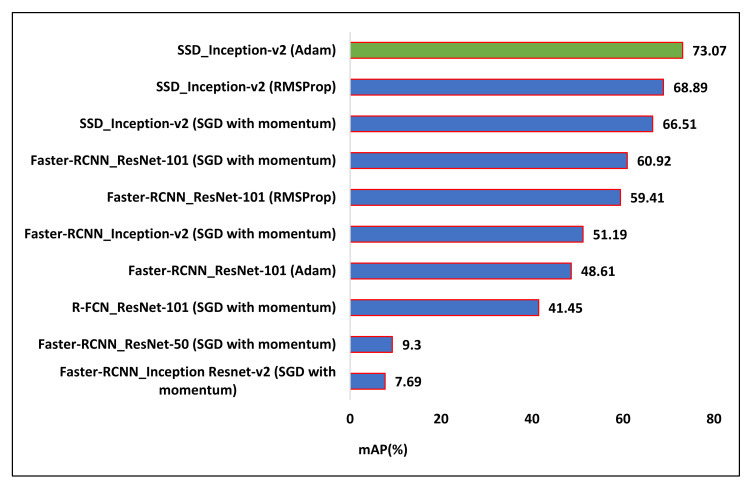
Summary of mean average precision (%) achieved by the DL meta-architectures with their respective optimizers (the best combination of DL meta-architecture and optimizer is shown by green bar).

After obtaining two best-suited DL meta-architectures, better optimization of the weight parameters was attempted by Adam and RMSProp optimization algorithms. Their learning rate is presented in Table 3. Table 4 presents the change in mAP for both the selected models. Some concluding remarks are provided as follows:

- The Faster-RCNN with the ResNet-101 model trained by Adam and RMSProp optimizers failed to improve its overall mAP as compared to the SGD (with momentum) optimizer.
- On the other hand, the SSD model achieved 66.51% mAP when it was trained by the momentum optimizer. Then, its mAP was increased by about 2.38% with the RMSProp optimizer. Further improvement of 3.39% in the mAP was observed when the weights of the SSD model were optimized by the Adam optimization algorithm.
- It is also noticed that when the SSD model was trained by Adam optimizer, the average precision of several leaf categories significantly improved, due to which the highest mAP of 73.07% was attained. The AP of classes such as Apple black rot, Apple cedar rust, Tomato early blight, and disease was increased to more than 50%. The AP of few other classes also improved (but still less than 50%) including Tomato target spot, Tomato bacterial spot, Potato late blight, Potato early blight, Pepper bacterial spot, and Peach bacterial spot. The AP of corn gray leaf spot class also improved, which was previously unsuccessful in providing a noticeable AP when the dataset was trained with the SGD with momentum and RMSProp optimizers. However, the further improvement in AP should be considered in future research.
- Figure 15 presents the change in AP for each class of the PlantVillage dataset when they were trained by the SSD model with all the three DL optimizers.
- A summary of the mAP achieved by DL meta-architectures trained with different optimization algorithms is presented in Figure 16.

## 4. Conclusions and Future Work

The main goal of this research was to perform the complex task of plant disease localization and classification in a single framework. In this regard, state-of-the-art deep learning meta-architectures including SSD, Faster RCNN, and RFCN models were trained and tested on 38 different classes of healthy/defected plant leaves. Moreover, an improvement in their performance was also attempted by better optimization of weight parameters through Adam and RMSProp optimizers. The SSD model trained with the feature extractor Inception-v2 attained the highest mean average precision as compared to the other DL meta-architectures. It achieved the best identification results by training through an Adam optimizer and attained 73.07% of mAP. All the healthy/diseased leaf classes were identified, which proves the novelty of the proposed approach. Practically, the successful detection of plant disease by DL technique would be useful to reduce the undesirable application of fungicide spray.

Few future recommendations for the research community are presented as follows:

- The trained and tested DL models’ pipeline, checkpoints, and weights can be reused as a transfer learning approach for upcoming researches related to plant disease detection.
- Various factors affecting the performance of best-suited DL architecture should be investigated such as data augmentation techniques, batch size, aspect ratios, etc.
- Although, all the classes of the PlantVillage dataset were identified by the proposed methodology; still, few of them achieved a lower average precision. Therefore, few modifications in DL networks can also be proposed in the future to further improve the mean average precision.
- This research could also be beneficial for several robotic systems to identify/classify healthy and unhealthy crops in real-time that would contribute to agricultural automation.

## Figures and Tables

**Table 1:** List of classes of PlantVillage dataset along with the cause of disease, annotation labels, number of training, validation, and testing images.

Classes of PlantVillage Dataset	Disease Cause	Annotation Label	Training Images	Validation Images	Testing Images
**Apple Scab**	Fungi	A_Scab	441	126	63
**Apple Black Rot**	Fungi	A_Blk_Rot	435	124	62
**Apple Cedar Rust**	Fungi	A_C_Rust	192	55	28
**Apple Healthy**	-	A_Healthy	1151	329	165
**Blueberry Healthy**	-	B_Healthy	1051	300	151
**Cherry Healthy**	-	Ch_Healthy	598	171	85
**Cherry Powdery Mildew**	Fungi	Ch_Mildew	736	210	106
**Corn (maize) Common rust**	Fungi	Corn_Rust	835	238	119
**Corn (maize) Healthy**	-	Corn_Healthy	813	233	116
**Corn (maize) Northern Leaf Blight**	Fungi	Corn_Blight	690	197	98
**Corn (maize) Gray leaf spot**	Fungi	Corn_Spot	360	102	52
**Grape Black Rot**	Fungi	G_Blk_Rot	826	236	118
**Grape (Black Measles)**	Fungi	G_Blk_Measles	968	277	138
**Grape Healthy**	-	Grp_Healthy	296	85	42
**Grape Leaf Blight (Isariopsis Leaf Spot)**	Fungi	Grp_Blight	753	215	108
**Orange Huanglongbing (Citrus greening)**	Bacteria	O_HLBing	3855	1101	551
**Peach Bacterial Spot**	Bacteria	Pec_Bact_Spot	1608	459	230
**Peach Healthy**	-	Pec_Healthy	252	72	36
**Pepper Bell Bacterial Spot**	Bacteria	Pep_Bact_Spot	698	199	100
**Pepper Bell Healthy**	-	Pep_Healthy	1034	297	147
**Potato Early Blight**	Fungi	Po_E_Blight	700	200	100
**Potato Healthy**	-	Po_Healthy	107	30	15
**Potato Late Blight**	Infection	Po_L_Blight	700	200	100
**Raspberry Healthy**	-	Ras_Healthy	260	74	37
**Soybean Healthy**	-	Soy_Healthy	3563	1018	509
**Squash Powdery Mildew**	Fungi	Sq_Powdery	1285	367	183
**Strawberry Healthy**	-	Straw_Healthy	319	91	46
**Strawberry Leaf Scorch**	Fungi	Straw_Scorch	776	222	111
**Tomato Bacterial Spot**	Bacteria	Tom_Bact_Spot	1488	426	213
**Tomato Early Blight**	Fungi	Tom_E_Blight	700	200	100
**Tomato Healthy**	-	Tom_Healthy	1114	318	159
**Tomato Late Blight**	Infection	Tom_L_Blight	1336	382	191
**Tomato Leaf Mold**	Fungi	Tom_L_Mold	667	190	95
**Tomato Septoria leaf Spot**	Fungi	Tom_Sept	1240	354	177
**Tomato Spider Mites**	Mite	Tom_Sp_Mite	1174	335	167
**Tomato Target Spot**	Fungi	Tom_Target	984	280	140
**Tomato Mosaic Virus**	Virus	Tom_Mosaic	262	74	37
**Tomato Yellow Leaf Curl Virus**	Virus	Tom_Curl	3750	1071	536

**Table 2:** List of base DL models with feature extraction methods along with mean average precision (mAP) on the Common Objects in Context (COCO) dataset.

Base Networks	Feature Extraction Methods	mAP (%) for COCO Dataset
**SSD**	Inception v2	24
**Faster-RCNN**	Inception v2	28
	ResNet-50	30
	ResNet-101	32
	Inception-ResNet v2	37
**R-FCN**	ResNet-101	30

**Table 3:** List of hyperparameters of the respective DL optimizers.

DL Optimizers	*lr*	Momentum	$\beta /\beta_{1}$	$\beta_{2}$	ξ
**SGD with Momentum (default)**	0.01	0.9	-	-	-
**SGD with Momentum (modified)**	0.0003	0.9	-	-	-
**Adam (default)**	0.001	-	0.9	0.999	1 × 10^−08^
**Adam (modified)**	0.0002	-	0.9	0.9997	1 × 10^−03^
**RMSProp (default)**	0.001	0.0	0.9	-	1 × 10^−08^
**RMSProp (modified)**	0.0004	0.0	0.95	-	1 × 10^−02^

**Table 4:** Summary of plant disease identification results indicating the Average Precision (AP) for each leaf class and the overall mAP for each DL meta-architecture. The hyphen (-) denotes the failed detection in the respective classes.

Annotated Class Labels	DL Meta-Architectures with Feature Extractors and Optimizers									
	R-FCN ResNet-101	Faster-RCNN						SSD Inception-v2		
		ResNet-50	Inception ResNet-v2	Inception-v2	ResNet-101					
	SGD with Momentum	SGD with Momentum	SGD with Momentum	SGD with Momentum	SGD with Momentum	RMSProp	Adam	SGD with Momentum	RMSProp	Adam
**A_Scab**	90.61	-	90.04	31.22	61.37	99.83	22.93	27.24	36.16	27.27
**A_Blk_Rot**	47.36	89.69	-	24.6	41.82	57.41	43.82	45.42	45.45	54.55
**A_C_Rust**	90.24	65.33	-	7.27	34.98	91.64	35.48	45.45	45.45	54.55
**A_Healthy**	90.85	99.97	2.19	75.91	43.42	6.71	18.13	90.91	100	90.91
**B_Healthy**	99.97	-	100	85.58	90	22.77	88.8	100	90.91	100
**Ch_Healthy**	79.22	-	-	87.32	75.69	99.84	63.66	90.86	90.91	90.91
**Ch_Mildew**	40.46	-	-	99.62	99.55	95.39	98.87	36.36	45.45	45.45
**Corn_Rust**	0.76	-	-	99.85	99.89	100	99.63	90.91	99.89	99.92
**Corn_Healthy**	-	-	-	100	99.96	76.13	90.87	90.91	90.91	90.91
**Corn_Blight**	42.57	-	-	51.01	66.34	93.53	96.09	45.45	54.55	54.29
**Corn_Spot**	0.31	-	-	1.15	20.56	31.83	10.95	0.73	5.62	33.13
**G_Blk_Rot**	7	-	-	1.16	53.9	53.87	0.05	71.26	72.73	72.73
**G_Blk_Measles**	0.09	-	-	100	100	100	99.61	100	90.91	99.87
**Grp_Healthy**	-	-	-	99.59	99.3	98.86	90.55	100	90.91	100
**Grp_Blight**	0.21	-	-	72.73	99.93	94.29	81.06	72.73	72.73	72.73
**O_HLBing**	12.74	-	-	99.99	90.91	98.14	90.91	90.91	90.91	90.91
**Pec_Bact_Spot**	0.28	-	-	14.23	40.23	74.16	-	9.09	18.14	27.23
**Pec_Healthy**	-	-	-	8.21	34.11	42.93	5.34	90.91	99.28	100
**Pep_Bact_Spot**	6.8	-	-	1.86	2.58	35.61	-	9.09	18.11	36.27
**Pep_Healthy**	50.95	-	-	6.45	2.11	19.08	2.33	90.91	90.8	90.91
**Po_E_Blight**	59.95	-	-	-	2.51	32	-	9.09	9.09	26.84
**Po_Healthy**	-	-	-	-	-	0.18	-	90.91	90.61	90.91
**Po_L_Blight**	94.77	-	-	-	-	1.79	-	29.16	43.81	44.92
**Ras_Healthy**	0.23	-	-	0.33	1.6	9.53	1.14	90.91	100	100
**Soy_Healthy**	88.11	-	-	26.03	59.43	9.6	3.35	90.91	90.13	90.91
**Sq_Powdery**	99.46	-	-	52.68	99.4	5.65	54.61	81.82	90.91	81.82
**Straw_Healthy**	99.3	-	-	53.33	18.07	1.1	62.53	100	90.91	100
**Straw_Scorch**	100	98.34	-	72.62	70.47	86.45	6.94	72.66	72.7	72.69
**Tom_Bact_Spot**	98.85	-	99.93	0.29	2.3	5.57	-	18.03	26.67	36.28
**Tom_E_Blight**	-	-	-	7.04	39.38	64.2	11.59	27.12	36.36	54.45
**Tom_Healthy**	0.2	-	-	100	87.13	49.65	100	100	100	100
**Tom_L_Blight**	-	-	-	99.96	99.96	95.92	90.36	81.79	81.72	90.77
**Tom_L_Mold**	3.87	-	-	96.21	98.7	99.55	82.21	45.41	63.6	63.64
**Tom_Sept**	-	-	-	99.56	95.24	100	99.83	90.86	90.88	90.91
**Tom_Sp_Mite**	85.12	-	-	98.52	98.06	98.73	99.9	90.88	90.88	90.91
**Tom_Target**	1.12	-	-	61.56	99.98	83.77	96.41	35.81	35.24	45.4
**Tom_Mosaic**	98.15	-	-	9.8	85.98	22.17	-	72.73	54.55	63.64
**Tom_Curl**	85.48	-	-	99.68	99.98	99.86	100	100	100	100
**mAP (%)**	41.45	9.30	7.69	51.19	60.92	59.41	48.63	66.51	68.89	73.07
